# Probiotic Yeasts and *Vibrio anguillarum* Infection Modify the Microbiome of Zebrafish Larvae

**DOI:** 10.3389/fmicb.2021.647977

**Published:** 2021-06-23

**Authors:** Orlando Vargas, María Soledad Gutiérrez, Mario Caruffo, Benjamín Valderrama, Daniel A. Medina, Katherine García, Angélica Reyes-Jara, Magaly Toro, Carmen G. Feijóo, Paola Navarrete

**Affiliations:** ^1^Laboratory of Microbiology and Probiotics, Institute of Nutrition and Food Technology (INTA), University of Chile, Santiago, Chile; ^2^ANID – Millennium Science Initiative Program - Millennium Nucleus in the Biology of the Intestinal Microbiota, Santiago, Chile; ^3^Laboratorio Inmunologia en Peces, Facultad de Ciencias de la Vida, Universidad Andres Bello, Santiago, Chile; ^4^Laboratorio de Biotecnología Aplicada, Facultad de Medicina Veterinaria, Universidad San Sebastián, Puerto Montt, Chile; ^5^Facultad de Ciencias de la Salud, Instituto de Ciencias Biomédicas, Universidad Autónoma de Chile, Santiago, Chile

**Keywords:** probiotic yeasts, *Debaryomyces hansenii*, *Yarrowia lipolytica*, *Vibrio anguillarum*, *Danio rerio* larvae, *Tg(Bacmpx:GFP)^*i114*^*, microbiome, neutrophils

## Abstract

The host microbiome plays an essential role in health and disease. Microbiome modification by pathogens or probiotics has been poorly explored especially in the case of probiotic yeasts. Next-generation sequencing currently provides the best tools for their characterization. *Debaryomyces hansenii* 97 (*D. hansenii* 97) and *Yarrowia lipolytica* 242 (*Y. lipolytica* 242) are yeasts that protect wildtype zebrafish (*Danio rerio*) larvae against a *Vibrio anguillarum* (*V. anguillarum*) infection, increasing their survival rate. We investigate the effect of these microorganisms on the microbiome and neutrophil response (inflammation) in zebrafish larvae line *Tg(Bacmpx:GFP)*^*i*114^. We postulated that preinoculation of larvae with yeasts would attenuate the intestinal neutrophil response and prevent modification of the larval microbiome induced by the pathogen. Microbiome study was performed by sequencing the V3-V4 region of the 16S rRNA gene and prediction of metabolic pathways by Piphillin in conventionally raised larvae. Survival and the neutrophil response were both evaluated in conventional and germ-free conditions. *V. anguillarum* infection resulted in higher neutrophil number in the intestinal area compared to non-infected larvae in both conditions. In germ-free conditions, infected larvae pre-inoculated with yeasts showed fewer neutrophil numbers than infected larvae. In both conditions, only *D. hansenii* 97 increased the survival of infected larvae. Beta diversity of the microbiota was modified by *V. anguillarum* and both yeasts, compared to non-inoculated larvae. At 3 days post-infection, *V. anguillarum* modified the relative abundance of 10 genera, and pre-inoculation with *D. hansenii* 97 and *Y. lipolytica* 242 prevented the modification of 5 and 6 of these genera, respectively. Both yeasts prevent the increase of *Ensifer* and *Vogesella* identified as negative predictors for larval survival (accounting for 40 and 27 of the variance, respectively). In addition, yeast pre-inoculation prevents changes in some metabolic pathways altered by *V. anguillarum*’s infection. These results suggest that both yeasts and *V. anguillarum* can shape the larval microbiota configuration in the early developmental stage of *D. rerio*. Moreover, modulation of key taxa or metabolic pathways of the larval microbiome by yeasts can be associated with the survival of infected larvae. This study contributes to the understanding of yeast–pathogen–microbiome interactions, although further studies are needed to elucidate the mechanisms involved.

## Introduction

The host microbiota participates in several physiological processes such as development, digestion, metabolism, immune stimulation, and neurological functions. In addition, it contributes to protect the host from invasion by exogenous microorganisms (i.e., colonization resistance) (Stecher et al., 2013; Yurist-Doutsch et al., 2014). Indeed, mice reared germ-free or treated with antibiotics are more susceptible to infections (Bäumler and Sperandio, 2016), and some microbiota configurations are more associated with infection resistance (Willing et al., 2011; Kampmann et al., 2016). Thus, one of the main challenges to combat infectious diseases is to understand how this microbial community can be manipulated into more resistant phenotypes, for example through probiotics.

Probiotics are defined as “live microorganisms that, when administered in adequate amounts, confer a health benefit on the host” (Hill et al., 2014). They were initially used to prevent or treat diarrhea by maintaining healthy gut microbiota and avoid its modification provoked by enteropathogens (dysbiosis). Effects of probiotics, especially *Bifidobacterium* and *Lactobacillus*, on microbiota composition have been largely studied by quantifying some specific bacterial populations through qPCR, fluorescence *in situ* hybridization, or other approaches (Barc et al., 2008; Swidsinski et al., 2016). Nowadays, new sequencing technologies have been developed to describe in further detail the bacterial communities present in the host microbiota and they have been recently used to explore the effect of probiotics on microbiota (Everard et al., 2014; Kabbani et al., 2017; Zmora et al., 2018). However, few studies describing their effect in an infection context using new sequencing approaches have been conducted (Villar-García et al., 2017; Cárdenas et al., 2020; Khan and Chousalkar, 2020).

Studies aimed to reveal mechanisms behind probiotic effects on infectious diseases rely on gnotobiotic animal models, such as mice, but this is a costly and labor-intensive approach. New vertebrate models—such as zebrafish (*Danio rerio*)—have become a useful tool to study pathogen–host interaction and, more recently, to explore mechanisms associated with probiotic effects (Rendueles et al., 2012; Caruffo et al., 2015, 2016; He et al., 2017; Qin et al., 2017; Pérez-Ramos et al., 2018; Yi et al., 2019; Chin et al., 2020; Dey and Kang, 2020). Zebrafish is a well-recognized animal model to study human development and disease (Vacaru et al., 2014), and, due to its phylogenetic proximity, it has been used as a model for cultured fish species (Dahm and Geisler, 2006; Ulloa et al., 2011). The functional organization of the zebrafish gut is similar to humans (Flores et al., 2020). Its gut is composed of absorptive enterocytes, mucus-producing goblet cells, and an enteroendocrine lineage (Flores et al., 2020) and its microbiota harbored the same phyla identified in mice and human (Rawls et al., 2004). In addition, a common response between the intestine of zebrafish and mice to their microbiota has been identified including stimulation of epithelial proliferation, promotion of nutrient metabolism, and innate immune responses (Rawls et al., 2004). Due to the transparency of larvae and the development of transgenic lines with fluorescently labeled immune cell populations, it is possible to *in vivo* visualize not only the pathogen but also the host response to infection (Renshaw et al., 2006; Meijer and Spaink, 2011; Meijer et al., 2014). For example, the transgenic line *Tg(Bacmpx:GFP)*^*i*114^, which carries labeled neutrophils that are fluorescently green, can be used to study the effect of pathogens on this specific cell population since they are the first group of leukocytes recruited to infection sites (Harvie and Huttenlocher, 2015).

Most probiotic strains are bacteria; however, yeasts have several attributes that may present them as good probiotic candidates (Sen and Mansell, 2020). Yeasts are part of the gut microbiota of animals including humans, but their role as a beneficial component has been less explored. They are naturally resistant to antibacterial agents and contain immune-stimulant molecules in their cell walls, such as β-glucans (Navarrete and Tovar-Ramírez, 2014). Some strains have shown antagonistic activities against pathogens (Hatoum et al., 2012) and trophic effects (Buts, 2009; Pais et al., 2020). These studies have been mainly focused on the strain *Saccharomyces boulardii* CNCM I-745 (also known as CBS 5926), which was isolated from lychee fruit (Czerucka et al., 2007). A significant amount of evidence showed that this probiotic yeast reduced the risk of antibiotic-associated diarrhea (Szajewska and Kołodziej, 2015). Other yeast strains, isolated from the intestine of healthy fish have shown a promising effect on survival and the activity of digestive enzymes, immune system, and growth of larval and adult fish (Navarrete and Tovar-Ramírez, 2014; Angulo et al., 2020). However, the interaction between intestinal commensal yeasts with bacterial pathogens, other members of the gut microbiota, and the host has remained largely unexplored in all vertebrates. In previous studies, we isolated two yeast strains, *Debaryomyces hansenii* 97 (*D. hansenii* 97) and *Yarrowia lipolytica* 242 (*Y. lipolytica* 242) from the intestinal microbiota of healthy fish (Raggi et al., 2014). These yeast strains have the potential for application in the aquaculture sector, considering their fish origin and the health benefits reported in the zebrafish model. They protected conventionally raised and germ-free wild type (Tab 5) zebrafish larvae against a *Vibrio anguillarum* infection by increasing their survival (Caruffo et al., 2015). Both yeast strains reduced the pathogen concentration in the host and prevented the up-regulation of inflammatory cytokines (*il1b*, *tnfa*, *c3*, *mpx*) (Caruffo et al., 2016). Larvae challenged with *V. anguillarum* showed an increase in neutrophil migration outside the hematopoietic region (Caruffo et al., 2015). However, the contribution of these probiotic yeasts to modulate neutrophil response and microbiome composition in the context of bacterial infection has not been addressed.

The transgenic zebrafish line *Tg(Bacmpx:GFP)*^*i*114^ was used to investigate the effect of two potential probiotic yeasts and the pathogen *V. anguillarum* on the microbiome composition and neutrophil response. The study was conducted in the early development stage when larvae did not depend on external feeding but only from nutrients derived from the yolk. We also investigated whether probiotic yeasts *D. hansenii* 97 and *Y. lipolytica* 242 can affect neutrophil recruitment to the intestine, one of the entry points of *V. anguillarum*. In addition, we explored whether both yeasts prevented the changes in microbiome composition exerted by *V. anguillarum* infection. The microbiome study was conducted by the sequencing V3–V4 region of the 16S rRNA gene and prediction of the metabolic pathways was performed by Piphillin. We postulated that the pre-inoculation of larvae with each yeast would attenuate the neutrophil response and prevent modification of the larval microbiome induced by the pathogen.

## Materials and Methods

### Zebrafish Maintenance

Adult zebrafish were maintained at the fish facility of the Universidad Andres Bello, following standard protocols (Westerfield, 2000). *Tg(Bacmpx:GFP)*^*i*114^ larvae were used in this study (Renshaw et al., 2006). A culture of conventionally raised larvae (with their microbiota) was performed with embryos obtained by natural spawning and cleaned immediately, removing detritus and unfertilized eggs, and then maintained at 28°C in E3 medium (5 mM NaCl, 0.17 mM KCl, 0.33 mM CaCl_2_, 0.33 mM MgSO_4_, pH 7.0) using sterile Petri dishes (100 embryos/dish) with daily cleaning. The 75% of the E3 medium volume was replaced daily with sterile E3 medium to avoid waste accumulation and oxygen limitation. Germ-free *Tg(Bacmpx:GFP)*^*i*114^ larvae were obtained as previously reported (Pham et al., 2008) with some modifications. Briefly, embryos obtained by natural spawning were collected in sterile E3 medium with antibiotics [kanamycin (5 μg/mL), ampicillin (200 μg/mL), ceftazidime (200 μg/mL), amphotericin B (250 ng/mL), and chloramphenicol (20 μg/mL)] until 7 h post fecundation (hpf). Eggs were then successively washed in a povidone-iodine (0.01%) and a sodium hypochlorite solution (0.003%). Then, eggs were distributed in groups of 100 in sterile Petri dishes containing E3 medium with antibiotics. Sterility of axenic larvae and E3 medium was daily tested (Milligan-Myhre et al., 2011). In brief, samples of E3 medium and larvae were plated in Trypticase Soy Agar and incubated aerobically overnight at 28°C. The manipulation of larvae for stereoscope analysis was done in anesthetized fish with tricaine (20 mg/L). Because zebrafish larvae can survive with nutrients derived from the yolk, they were reared without feeding until 8 dpf (Bates et al., 2006). Larvae were euthanized with an overdose of tricaine (200 mg/L) when necessary. The protocols with zebrafish larvae were approved by the local and central Committee for Ethics of Animal Experiments from INTA and the University of Chile (FCYT1-18-PN).

### *Vibrio anguillarum* and Yeast Cultures

The pathogenic strain *V. anguillarum* PF4 was cultured in Trypticase Soy Broth (TSB) with 0.5% NaCl for 4 h at 28°C to reach log-phase (Caruffo et al., 2015, 2016). *V. anguillarum* cells were then collected by centrifugation at 10,000 × *g* for 5 min, washed twice with sterile E3 medium, and resuspended in the same medium to inoculate larvae, as described below. *D. hansenii* 97 and *Y. lipolytica* 242 yeasts were cultured in YPD broth (1% yeast extract, 2% peptone, and 2% dextrose with 0.05% chloramphenicol) at 28°C for 24 h with constant agitation. Then, yeast cells were centrifuged at 5,000 × *g* for 2 min, washed twice with sterile E3 medium, and resuspended in the same medium to inoculate larvae, as described below. The concentration of microorganisms inoculated in larvae was evaluated daily by plating serial ten-fold dilution of homogenized larvae in YPD agar with 0.05% chloramphenicol and CHROMagar *Vibrio* for yeast and *V. anguillarum*, respectively.

### Zebrafish Assay Experimental Design

Three days post-fertilization (dpf) *Tg(Bacmpx:GFP)*^*i*114^ larvae were randomly distributed in six groups in triplicate. The six groups were: (1) control group: non-inoculated control larvae, (2) Dh97 group: larvae inoculated with *D. hansenii* 97 at 4 dpf, (3) Yl242 group: larvae inoculated with *Y. lipolytica* 242 at 4 dpf, (4) Va group: larvae challenged with *V. anguillarum* at 5 dpf, (5) Dh97 + Va group: larvae inoculated with *D. hansenii* 97 at 4 dpf and challenged with *V. anguillarum* at 5 dpf, and (6) Yl242 + Va group: larvae inoculated with *Y. lipolytica* 242 at 4 dpf and challenged with *V. anguillarum* at 5 dpf (Supplementary Figure 1). Larvae were inoculated by immersion with each microorganism. The yeast and pathogen concentrations were adjusted to 5 × 10^6^ yeast/mL and 10^7^ *V. anguillarum*/mL, respectively. Larvae were maintained for 20 min with each microorganism concentration, then these were washed and maintained in sterile E3 medium. Larval mortality was daily recorded. Analysis of microbiota was performed in conventionally raised larvae. The neutrophil quantification and analysis of survival were performed in germ-free and conventionally raised larvae. To avoid cross-contamination, the handling of Petri dishes containing larvae from different groups was performed in a cell culture hood using sterile techniques (Pham et al., 2008). The efficacy of the germ-free condition and infection model, as well as safety of probiotic yeasts in each replicate, was validated according to the previously published data (Pham et al., 2008; Caruffo et al., 2015, 2016).

### Identification of Larval Microbiota Composition by Sequencing the 16S rRNA Gene

The identification of the bacterial microbiota of larvae was performed in conventionally-raised larvae (with their microbiota) in all groups at 8 dpf, corresponding to 3 days post *V. anguillarum* infection (Supplementary Figure 1). First, eight samples were taken from each group: 6 individuals and 2 pooled larvae (composed of 5 individuals each). Larvae were washed in sterile PBS, then homogenized in TE buffer pH 8.0 (10 mM Tris-HCl, 1 mM EDTA) and immediately stored at −20°C until DNA extraction. The Wizard^®^ Genomic DNA Purification (Promega) kit was used for DNA extraction in all samples, using sterile PBS as the negative control, and Libraries preparation and sequencing were performed as previously described (Morales et al., 2016) in the Roy J. Carver Biotechnology Center at the University of Illinois at Urbana-Champaign, Champaign, IL, United States. Briefly, the V3–V4 region of the 16S rRNA gene was amplified with the primers V3_F341_N (5′-CCTACGGGNGGCWGCAG-3′) and V4_R805R (5′-GACTACHVGGGTATCTAATCC-3′) (Klindworth et al., 2013) using the Fluidigm system (Fluidigm, South San Francisco, CA, United States). Amplicons were sequenced on a MiSeq Illumina platform (Illumina, San Diego, CA, United States), generating paired-end reads (2 × 300 nt). Demultiplexes and barcode-depleted sequences were delivered from sequencing services (W. M. Keck Center for Comparative and Functional Genomics, University of Illinois, United States).

Paired-end sequences were imported to R statistical language v3.5.2 (R Core Team, 2020) to be processed following the DADA2 v1.6 pipeline (Callahan et al., 2016) on RStudio environment. Briefly, the Fastq sequences were inspected for quality profile, filtered, and trimmed. The obtained files were used to learn the error rates and reduce the level of single-nucleotide differences over the sequenced region amplified. Then, the files were merged and a sequence table was built to remove chimera artifacts resulting from amplification. The taxonomy assignment was performed using the SILVA SSU database v138 (Quast et al., 2013). Non-rarefactioned data were used to build a phyloseq object (McMurdie and Holmes, 2013) for abundance and alpha and beta diversity estimations. The Microbiome package was used to estimate core diversity (Lahti et al., 2017). Finally, all the data were represented using the ggplot2 library (Ginestet, 2011).

### Neutrophil Quantification

Neutrophils are the first immune cells to arrive upon an insult due to physical damage or pathogen infection. There is also evidence that this increase in neutrophil number correlates with an increase in proinflammatory cytokines (Hedrera et al., 2013). Therefore, we used neutrophil quantification as a tool to assess the degree of inflammation in intestinal tissue (Ulloa et al., 2016; Coronado et al., 2019; Solis et al., 2020). Due to the transparency of larvae and the development of transgenic lines with fluorescently labeled immune cell populations, such as *Tg(Bacmpx:GFP)*^*i*114^, that carry labeled neutrophils that are fluorescently green, we will *in vivo* visualize the host response to infection (Harvie and Huttenlocher, 2015). Larvae were anesthetized with tricaine (20 mg/L) and positioned laterally to quantify the number of neutrophils present in the intestine, at 1 days post-challenge (corresponding to 6 dpf) (Supplementary Figure 1). Five larvae from each triplicate (15 larvae per group) were sampled and visualized using a fluorescence stereomicroscope Leica MZ10F at 8× objective (Leica, Germany) equipped with a green fluorescent filter ET GFP. Neutrophils quantification was performed in conventionally raised and germ-free larvae.

### Metabolic Pathways Inference

Metabolic pathways present in each condition were inferred from 16S rRNA sequences with Piphillin (Iwai et al., 2016) since it outperforms similar available tools (Narayan et al., 2020). The reference database used was KEGG (Narayan et al., 2020), the May 2020 version, and the identity cutoff was settled at 99%. The KEGG database is structured in different hierarchy levels. The highest level corresponds to seven “Main processes,” which in turn are composed of “General processes” and those included in “metabolic pathways.” In this study, we analyzed all “General processes” and their subsequent “metabolic pathways,” except those included in Organismal systems (which includes metabolic pathways not described in bacteria, such as digestive, circulatory, endocrine system, etc.), human diseases (cancer, cardiovascular or neurodegenerative diseases, etc.), and Drug development (nervous system agents, antineoplastics, etc.). We included the Main processes: Metabolism, Genetic Information Processing, Environmental Information Processing, and the Cellular Processes. Piphillin’s output was analyzed and formatted with the KEGGREST (Tenenbaum, 2020) R package, and statistical testing of differences in metabolic paths between groups was conducted with DESeq2 package (Love et al., 2014). Wald’s test with Benjamini–Hochberg FDR correction (alpha = 0.01) was applied in each contrast.

### Statistical Analysis

Statistical analysis was performed using the software GraphPad Prism 8 (Graphpad Software, Inc.) and R statistical language v3.5.2. Survival curves and group differences were analyzed using Kaplan–Meier and Log-rank test, respectively. Unpaired Mann–Whitney test was applied to detect significant differences in microbial counts (log_10_ CFU/larvae) for each group between conventional and germ-free conditions. The Kruskal–Wallis test and Dunn’s test with correction for multiple comparisons using statistical hypothesis testing (GraphPad) were used to identify significant differences in microbial counts (log_10_ CFU/larvae), the number of neutrophils/intestine, alpha diversity indices, and the relative abundance of phyla and genera between groups. Linear discriminant analysis effect size (LEfSe) was performed to identify which genera among those statistically different will explain the greatest proportion of differences between groups (Segata et al., 2011). A threshold of four was considered for the logarithmic LDA score. Spearman’s rank correlation coefficients were calculated to determine the correlation between survival and the relative abundance of all genera. A stepwise multiple regression analysis was performed to explore the best bacterial genera predicting survival from those showing significant correlation. Genera with a variance inflation factor (VIF) equal to or greater than five were excluded from the analysis. The significance level was set at *p* < 0.05. Wald’s test with Benjamini–Hochberg FDR correction (alpha = 0.01) was applied to detect differences between inferred metabolic pathways within groups.

## Results

### Effect of *V. anguillarum* and Yeasts on the Survival of *Tg(Bacmpx:GFP)*^*i*114^ Larvae

**FIGURE 1:**
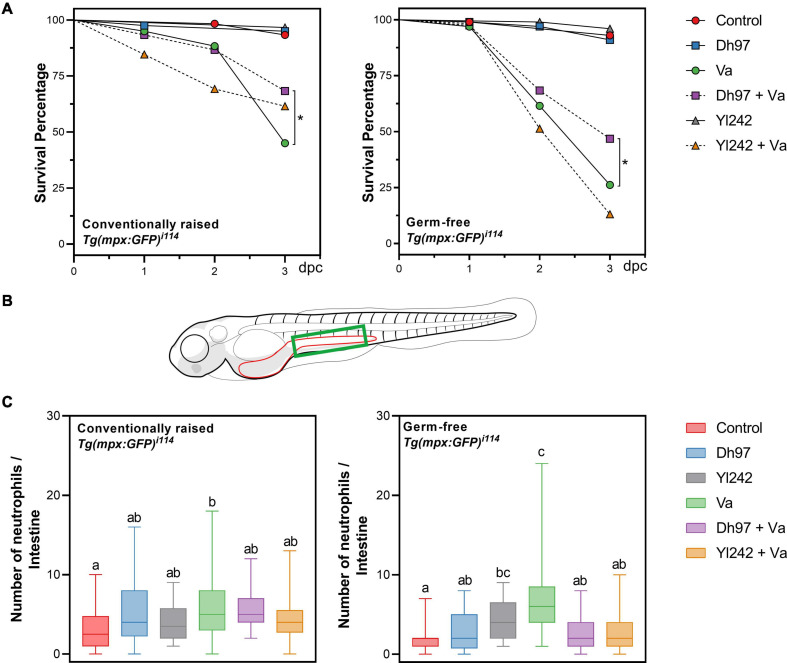
Survival and neutrophil counts in conventionally raised (CONV-R) and germ-free *Tg(Bacmpx:GFP)*^*i*114^ larvae inoculated with yeasts and/or *Vibrio anguillarum*. **(A)** Survival rate of germ-free and conventionally raised larvae (CONV-R) (Kaplan–Meier curves). Statistically significant differences were determined with a log-rank test (^∗^*p* < 0.05). **(B)** Representation of zebrafish larvae with part of the gastrointestinal tract highlighted in red. Neutrophil counts were performed in the green area. **(C)** Neutrophil counts in germ-free and conventionally raised *Tg(Bacmpx:GFP)*^*i*114^ zebrafish larvae. Data show box and whisker plot: each box represents the first to third quartiles, center bar the median, and whiskers the maximum and minimum of each dataset. Different letters denote *p* < 0.05, Kruskal–Wallis followed by Dunn’s test. Control group: non-inoculated larvae. Dh97 and Yl242 groups: larvae inoculated by immersion with 5 × 10^6^ CFU/mL of *D. hansenii* 97 or *Y. lipolytica* 242, at 4 dpf. Va group: larvae challenged at 5 dpf by immersion with 10^7^ CFU/mL of *V. anguillarum*. Dh97 + Va and Yl242 + Va groups: larvae inoculated by immersion with the respective yeast at 4 dpf and challenged with *V. anguillarum* at 5 dpf. Data represent three independent experiments.

We evaluated the effect of *V. anguillarum* infection (at 5 dpf) and pre-inoculation of both yeasts (at 4 dpf) on the survival rate of *Tg(Bacmpx:GFP)*^*i*114^ larvae (Figure 1A). After 3 days, the survival rate of larvae infected with *V. anguillarum* (Va group) was significantly lower than the survival of non-infected controls, in conventional (45 vs. 93.3%) and germ-free (26.2 vs. 93%) conditions. The survival of infected larvae was significantly lower in the germ-free (26.2%) condition compared to those raised in the conventional condition (45%) (*p* < 0.05), highlighting the protective effect of larval microbiota in this host. Conventional larvae pre-inoculated with yeasts and then infected with *V. anguillarum* (Dh97 + Va and Yl242 + Va) showed a higher survival rate compared to infected larvae (Va group), but this was only significant for *D. hansenii* 97 (*p* < 0.05) (Figure 1A). Similarly, only infected larvae pre-inoculated with *D. hansenii* 97 (Dh97 + Va) showed higher survival compared to infected larvae (Va group) (*p* < 0.05) in the germ-free condition (Figure 1A). This indicates that the protection of *Tg(Bacmpx:GFP)*^*i*114^ larvae against *V. anguillarum* depends on the host microbiota and yeast strain.

### Concentration of *V. anguillarum* and Yeasts in *Tg(Bacmpx:GFP)*^*i*114^ Larvae

The viability and persistence of each inoculated microorganism in larvae was daily determined by culturing homogenized larvae on selective agar media (Supplementary Tables 1, 2). *V. anguillarum* reached about 4 log_10_ CFU/larvae shortly after being inoculated at 5 dpf in germ-free and conventional conditions (Supplementary Table 1). Then, from 6 to 7 dpf, the pathogen persisted and reached a significantly higher concentration in germ-free larvae compared to those raised under conventional conditions (*p* < 0.05). Pre-inoculation of larvae with both yeasts did not affect the *V. anguillarum* concentration throughout the experiment in the conventional condition. In the germ-free condition only, larvae pre-inoculated with *D. hansenii* 97 were 1 log_10_ lower in pathogen concentration at 5 dpf than infected larvae (Va group) (*p* < 0.05). Yeast concentrations in larvae were strain-dependent and higher numbers were reached for *D. hansenii* 97 than for *Y. lipolytica* 242 (Supplementary Table 2). After yeast inoculation, at 4 dpf, the concentration of *Y. lipolytica* 242 reached approximately 3 log_10_ CFU/larvae in germ-free and conventional conditions, whereas the counts reach values of about 1 log_10_ CFU/larvae higher (*p* < 0.05) for *D. hansenii* 97. *Y. lipolytica* 242 counts in groups Yl242 and Yl242 + Va were lower for conventionally raised larvae, compared to germ-free (*p* < 0.05) at day 5 dpf. The counts of *D. hansenii* 97 were lower in the germ-free larvae for the Dh97 + Va group, only at 6 dpf (*p* < 0.05).

### *V. anguillarum*-Infected Larvae Preinoculated With *Y. lipolytica* 242 and *D. hansenii* 97 Show Less Neutrophil Than *V. anguillarum* Infected Larvae, in Germ-Free Condition

The neutrophil number in the intestinal region in response to *V. anguillarum* infection was determined using the transgenic line *Tg(Bacmpx:GFP)*^*i*114^ (Figures 1B,C). We focused on this anatomical area because the gastrointestinal tract has been reported as one of the main entry pathways for this pathogen (Caruffo et al., 2015; Oyarbide et al., 2015). One day after *V. anguillarum* challenge (Va group), a higher number of neutrophils present in the intestine of challenged larvae was observed compared to non-challenged larvae (control) in conventional and germ-free conditions (*p* < 0.05); indicating the trigger of an immune response against *V. anguillarum*. A higher number of neutrophils was also observed in larvae inoculated with *Y. lipolytica* 242 compared to non-inoculated ones, in the germ-free condition (*p* < 0.05). The infected larvae pre-inoculated with each yeast showed a lower neutrophil count than the Va group (*p* < 0.05) in the germ-free condition.

### Analysis of Larval Microbiota Composition

The bacterial communities were analyzed by sequencing the V3–V4 region of 16S rRNA gene from the six groups of conventionally raised larvae. We sequenced eight samples (six individuals and two pools of five larvae) from each group. A total number of 2,378,460 raw reads was obtained with an average value of 49,551 ± 20,977 reads per sample. After removing sequences with a Phred quality score below 30 and trim the base pairs belonging to the library barcodes, we obtained a total of 2,125,435 (89.4% of initial) reads ranging from 7,029 to 107,733. Then, removing reads present in negative DNA extraction controls, chimeric sequences, eukaryotic reads, and non-assigned sequences; we obtained a total of 651 amplicon sequence variants (ASVs). The rarefaction curves tended the saturation plateau for each sample, which indicated that sequencing depth was reasonable to adequately describe the full microbiota diversity (Supplementary Figure 2). All the ASVs were merged for the same taxonomy assigned, to obtain a total of 91 taxa (Supplementary Table 3) of which 25 were the most abundant among groups (Supplementary Table 4). Finally, we evaluated whether individual and pooled samples had similar diversity. The result showed that both sample types from each group presented similar ASVs and alpha diversity (Supplementary Figure 3), therefore both were included in the analysis.

### Alpha Diversity of Microbiota of *Tg(Bacmpx:GFP)*^*i*114^ Larvae Was Not Affected by Yeasts or *V. anguillarum* Infection

**FIGURE 2:**
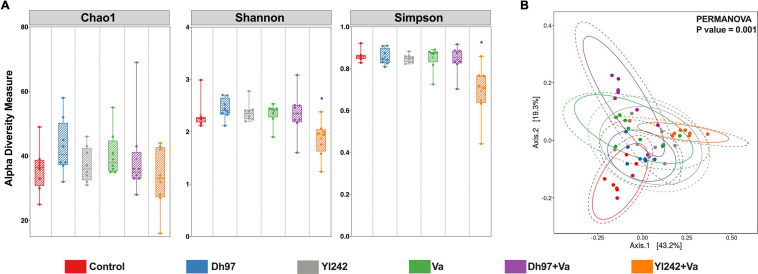
Bacterial diversity analyses of *Tg(Bacmpx:GFP)*^*i*114^ larvae inoculated with yeasts and/or *V. anguillarum*. **(A)** Alpha diversity indexes Chao1, Shannon, and Simpson. **(B)** Principal coordinate analysis (PCoA) based on weighted Unifrac distance matrix plot. Analysis of the bacterial microbiota was performed in *Tg(Bacmpx:GFP)*^*i*114^ zebrafish larvae at 8 days post fertilization (dpf), corresponding to 3 days post *V. anguillarum* infection. Control group: non-inoculated conventionally raised larvae (CONV-R). Dh97 and Yl242 groups: CONV-R larvae inoculated by immersion with 5 × 10^6^ CFU/mL of *D. hansenii* 97 or *Y. lipolytica* 242, respectively, at 4 dpf. Va group: CONV-R larvae challenged at 5 dpf by immersion with 10^7^ CFU/mL of *V. anguillarum*. Dh97 + Va and Yl242 + Va groups: CONV-R larvae inoculated by immersion with both yeasts at 4 dpf and challenged with V. *anguillarum* at 5 dpf. ^∗^*p* < 0.05.

An analysis of alpha diversity with the estimators of community richness (Chao1), diversity (Shannon), richness, and evenness (Simpson) was calculated (Figure 2A). Exposure of larvae to each yeast and *V. anguillarum* did not significantly affect the microbial alpha diversity measures compared to the control group (Kruskal–Wallis and Dunn test, *p* > 0.05). However, infected larvae pre-inoculated with *Y. lipolytica* 242 (Yl242 + Va group) showed lower values for the Shannon and Simpson indices compared to infected larvae (Va group) and lower Simpson index values compared to the control group (*p* < 0.05).

### Both Yeasts and *V. anguillarum* Infection Altered the Beta Diversity of Microbiota of *Tg(Bacmpx:GFP)*^*i*114^ Larvae and the Relative Abundance of Some Bacterial Genera

Principal coordinate analysis (PCoA) based on a weighted Unifrac distance matrix showed that the structure of bacterial microbiota was significantly different between the six groups (PERMANOVA; *p* = 0.001) (Figure 2B). The infection by *V. anguillarum* significantly affected the microbiota of larvae, as well as the inoculation with each yeast (group Dh97 and Yl242) (*p* = 0.001). Preinoculation of infected larvae with yeasts did not prevent the modification of the microbiota exerted by the pathogen since microbiota of groups Dh97 + Va and Yl242 + Va were significantly different from the control larvae (*p* = 0.001).

**FIGURE 3:**
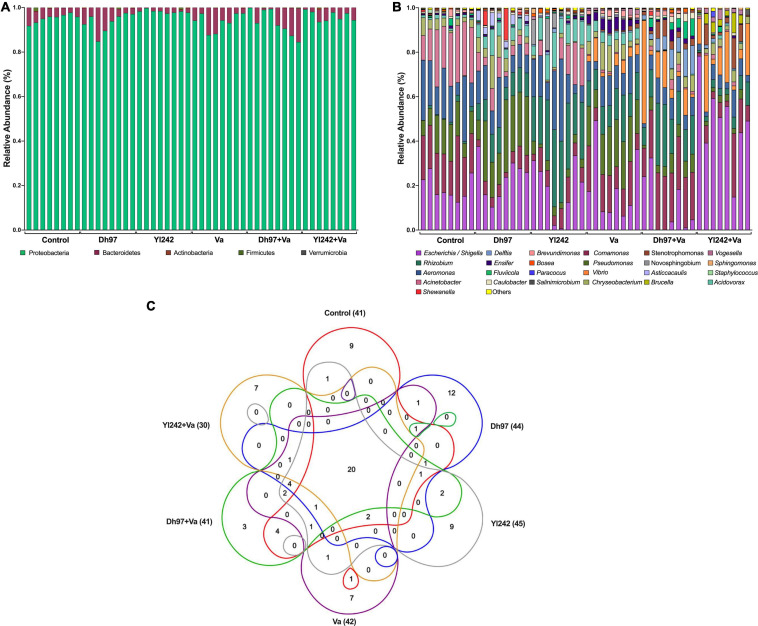
Bacterial composition of *Tg(Bacmpx:GFP)*^*i*114^ larvae inoculated with yeasts and/or *Vibrio anguillarum*. **(A)** Phylum level, **(B)** genus level, and **(C)** venn diagram showing the number of shared and unique genera among groups. Analysis of the bacterial microbiota was performed in *Tg(Bacmpx:GFP)*^*i*114^ zebrafish larvae at 8 days post fertilization (dpf), corresponding to 3 days post *V. anguillarum* infection. Control group: non-inoculated conventionally raised larvae (CONV-R). Dh97 and Yl242 groups: CONV-R larvae inoculated by immersion with 5 × 10^6^ CFU/mL of *D. hansenii* 97 or *Y. lipolytica* 242, respectively, at 4 dpf. Va group: CONV-R larvae challenged at 5 dpf by immersion with 10^7^ CFU/mL of *V. anguillarum*. Dh97 + Va and Yl242 + Va groups: CONV-R larvae inoculated by immersion with both yeasts at 4 dpf and challenged with *V. anguillarum* at 5 dpf.

The microbiota communities were composed of the phyla *Proteobacteria*, *Bacteroidetes*, *Firmicutes*, *Actinobacteria*, and *Verrucomicrobia* (Figure 3A). *Proteobacteria* and *Bacteroidetes* represented more than 98% of the relative abundance in all samples. The relative abundance of phyla was not affected by any microorganisms, except for the Yl242 group which showed a higher relative abundance of *Proteobacteria* compared to Dh97 and infected Va group (*p* < 0.05). This increase occurred at the expense of *Bacteroidetes* (*p* < 0.05). We identified 91 taxa at the level of genera in all groups, of which 41 were identified in the control group (Figure 3B and Supplementary Table 3). The inoculation of *D. hansenii* 97, *Y. lipolytica* 242, and *V. anguillarum* showed 12, 9, and 7 new genera identified, respectively, whereas 20 genera were common to all groups (Figure 3C). We also identified, exclusively in infected larvae preinoculated with yeasts, three new genera for the Dh97 + Va group, and seven genera for the Yl242 + Va group.

The bacteria shared among different individuals in the control group, i.e., the core microbiota, were identified because they are considered to be associated with health status. This core and the core of the other groups were calculated with different prevalence and abundance thresholds (Supplementary Figure 4). In the control group, if we consider 75% and 100% prevalence [genera present in 6 (75%) and 8 (100%) of 8 samples] and the low abundant taxa, we identified 13 (31%; 13/41) and 14 genera (34%; 14/41), respectively. The genera composing the core of control larvae (with prevalence of 75%) account for more than 99% of the relative abundance (Supplementary Table 4). The core of the control group was composed by *Acinetobacter* (21%), *Comamonas* (19.5%), *Escherichia*/*Shigella* (19.1%), *Aeromonas* (18.0%), *Pseudomonas* (11.9%), *Chryseobacterium* (3.8%), *Acidovorax* (1.2%), *Rhizobium* (1.1%), *Brevundimonas* (0.9%), *Ensifer* (0.7%), *Fluviicola* (0.7%), *Caulobacter* (0.6%), *Bosea* (0.3%), and *Vogesella* (0.15%) by decreasing order of relative abundance. The genera from the control core, present in the other groups, were identified, and more than 90% were shared with larvae inoculated with any microorganism, excepting for those infected larvae preinoculated with *Y. lipolytica* 242 (Yl242 + Va group), which shared 71.4% (10/14) taxa. Because of the lower prevalence (<75%) or lower relative abundance (<0.001%), four genera of the control core (*Fluviicola, Caulobacter, Brevundimonas*, and *Bosea*) were not considered as part of the core of the Yl242 + Va group.

Linear discriminant analysis Effect Size (LEfSe) was performed in order to identify which genera, among those statistically different, would explain the greatest proportion of differences between the communities (Supplementary Figure 5). *Acinetobacter* and *Aeromonas* were identified in the control group. Larvae inoculated with *D. hansenii* 97 were characterized by *Pseudomonas* and *Asticcacaulis* while *Y. lipolytica* 242 by *Rhizobium*, *Acidovorax*, *Sphingomonas*, and *Bosea*. Among challenged groups, in the Va group were identified *Chryseobacterium, Ensifer*, and *Brevundimonas*. Challenged larvae pre-inoculated with *D. hansenii* 97 (Dh97 + Va) were characterized by *Comamonas*, *Delftia*, *Caulobacter*, and *Fluvicola*, while the group pre-inoculated with Yl242 (Yl242 + Va) by the presence of *Escherichia, Shigella*, *Vibrio*, *Stenotrophomonas, Brucella*, and *Vogesella*.

**FIGURE 4:**
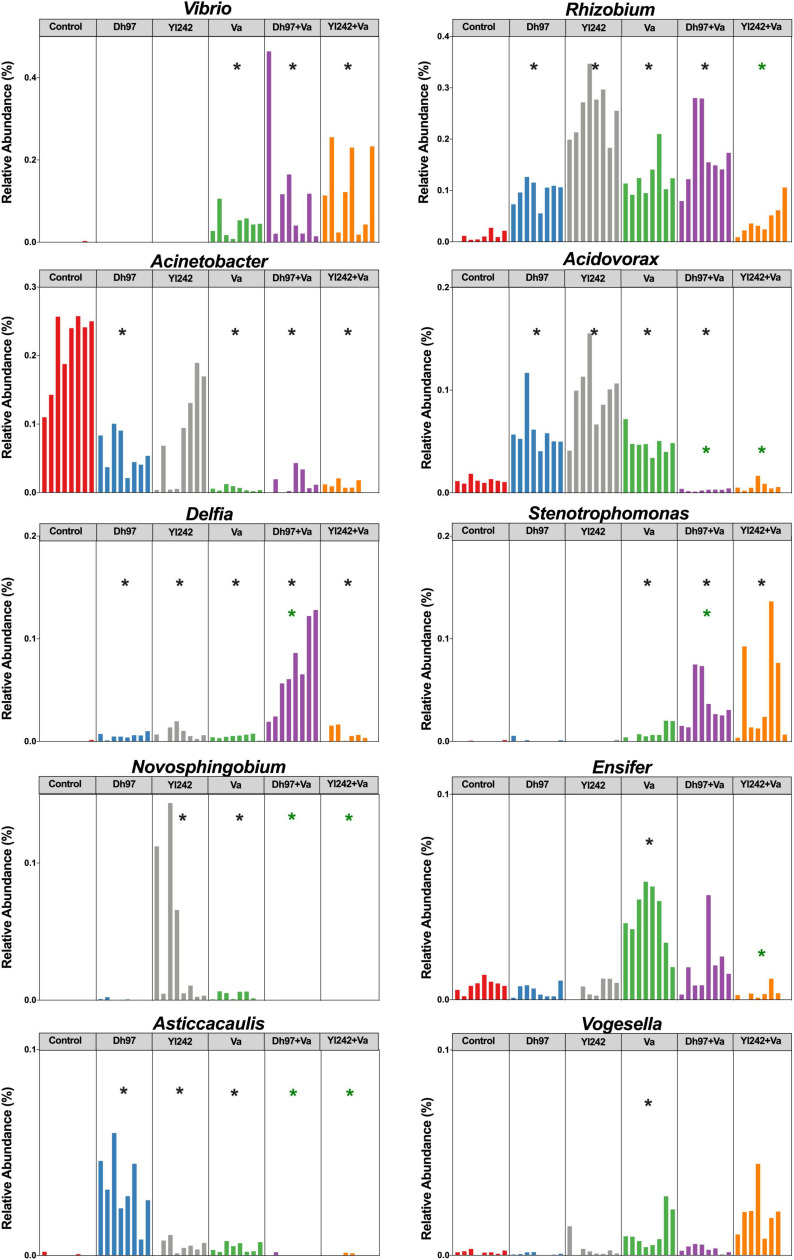
Relative abundance of bacterial genera affected by infection with *V. anguillarum*. Analysis of the bacterial microbiota was performed in *Tg(Bacmpx:GFP)*^*i*114^ zebrafish larvae at 8 days post fertilization (dpf), corresponding to 3 days post *V. anguillarum* infection. Control group: non-inoculated conventionally raised larvae (CONV-R). Dh97 and Yl242 groups: CONV-R larvae inoculated by immersion with 5 × 10^6^ CFU/mL of *D. hansenii* 97 or *Y. lipolytica* 242, respectively, at 4 dpf. Va group: CONV-R larvae challenged at 5 dpf by immersion with 10^7^ CFU/mL of *V. anguillarum*. Dh97 + Va and Yl242 + Va groups: CONV-R larvae inoculated by immersion with both yeasts at 4 dpf and challenged with *V. anguillarum* at 5 dpf. Significant differences between groups and the control group are marked with black asterisks. Green asterisks indicated significant differences between Dh97 + Va or Yl242 + Va with Va group (*p* < 0.05, Kruskal–Wallis followed by Dunn’s test).

The infection of larvae with *V. anguillarum* modified the relative abundance of 10 genera. It increased the relative abundance of *Vibrio*, *Rhizobium*, *Acidovorax*, *Delftia*, *Stenotrophomonas*, *Ensifer*, *Novosphingobium*, *Asticcacaulis*, and *Vogesella* (*p* < 0.05) and reduced *Acinetobacter* (*p* < 0.05) (Figure 4). The pre-inoculation of infected larvae with *D. hansenii* 97 and *Y. lipolytica* 242 prevented the increase of *Ensifer*, *Novosphingobium*, *Asticcacaulis, Acidovorax*, and *Vogesella* since their relative abundance was similar to control (*p* > 0.05). *Y. lipolytica* 242 also prevented the increase of *Rhizobium* (Control vs. Yl242 + Va*; p* > 0.05). Yeasts could not prevent the increase in *Vibrio*, *Delftia*, and *Stenotrophomonas* and the decrease of *Acinetobacter* in infected larvae (Control vs. Dh97 + Va; *p* < 0.05 and Control vs. Yl242 + Va; *p* < 0.05). Interestingly, the inoculation of yeasts alone induced modification in the abundance of some genera. *Y. lipolytica* 242 reduced *Pseudomonas* and increased *Novosphingobium* and *D. hansenii* 97, reduced *Acinetobacter* (*p* < 0.05). Both yeasts reduced the relative abundance of *Comamonas* (*p* < 0.05) and increased the abundance of *Rhizobium*, *Acidovorax*, *Delftia*, and *Asticcacaulis* (*p* < 0.05) compared to control.

We identified significant correlations between larval survival and the relative abundance of some bacterial genera through Spearman’s correlation analysis, at the end of the experiment (8 dpf) (Supplementary Table 5). Data showed that *Aeromonas*, *Acinetobacter*, *Acidovorax*, *Asticcacaulis*, *Bosea*, *Sphingomonas*, *Devosia*, and *Arsenicibacter* were positively correlated with survival (*p* < 0.05), whereas *Vibrio*, *Chryseobacterium*, *Stenotrophomonas, Ensifer*, *Brevundimonas*, *Vogesella*, *Novospirillum*, and *Xanthomonas* were negatively correlated (*p* < 0.05). A stepwise multiple regression analysis, incorporating these 16 genera, was conducted to predict larval survival. In total, 93% of the variance (*p* < 0.0001) was explained by eight genera specifically *Ensifer* (40%), *Vogesella* (27%), and *Vibrio* (15%) accounted for 82% of the variance, which were identified as negative predictors for survival. The pre-inoculation of infected larvae with *D. hansenii* 97 and *Y. lipolytica* 242 prevented the increase of *Ensifer* and *Vogesella*, as we stated above.

### Composition of the Metabolic Processes Identified Within the Control Group

An abundance of genes associated with the metabolic pathways was inferred from the 16S rRNA sequences with Piphillin software and the KEGG database. The analysis of metabolic processes composition for each sample within the control group showed low variability across samples (Supplementary Figure 6). The carbohydrate metabolism (0.146 ± 0.003), amino acid metabolism (0.138 ± 0.006), membrane transport (0.08 ± 0.002), cellular community–prokaryotes (0.07 ± 0.001), and metabolism of cofactor and vitamins (0.07 ± 0.001) processes were the most abundant functions in all control samples.

### Preinoculation With Yeasts Can Prevent Changes in Some Metabolic Pathways That Were Altered by *Vibrio anguillarum*’s Infection

An analysis with the DESeq2 R package was performed to determine whether the treatment induces changes in the abundance of genes associated with the metabolic pathways inferred from the 16S rRNA sequences through Piphillin. The contrasts were made between the control group and every other group, and differences were determined by Wald’s test with Benjamini–Hochberg FDR correction (alpha = 0.01). All groups showed differences with the control group detailed information of each one in Supplementary Table 6. Compared to the control group, larvae infected with *V. anguillarum* showed significant differences in the abundance of genes associated with the 29 metabolic pathways (Table 1). Interestingly, infected larvae pre-inoculated with yeasts showed no significant difference in some of those pathways when they were contrasted with the control group (Table 1). Of the 29 metabolic pathways altered by *V. anguillarum*, *D. hansenii* 97 avoided changes in 13, and *Y. lipolytica* 242 did so in 10. These metabolic pathways belong to carbohydrate-, energy-, amino acid-, cofactors-, and terpenoids/polyketides metabolism, as well as biosynthesis of other secondary metabolites, xenobiotics biodegradation and metabolism, transcription, folding, sorting, and degradation.

**TABLE 1:** KEGG metabolic pathways inferred by 16S rRNA sequencing showing significant differences between *Vibrio anguillarum* infected group (Va) and the control group, and the effect of the pre-inoculation of infected larvae with yeasts (Dh97 + Va and Yl242 + Va group) over these altered pathways.

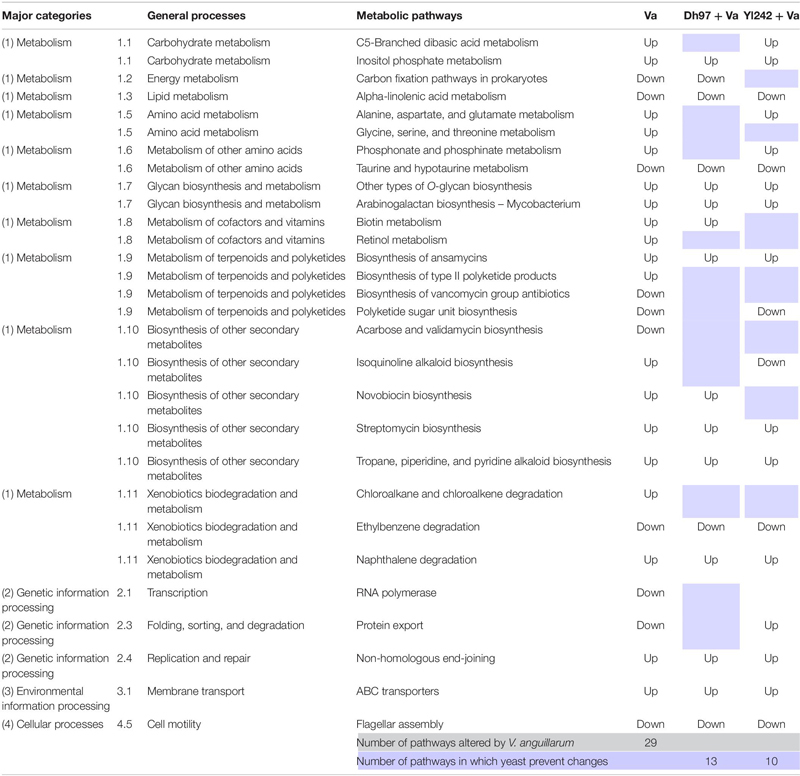

*“Up”: pathways showing a significant increase in the inferred genes of a particular pathway over the control group, “Down” is for the opposite. Highlighted cells indicate those pathways that show no differences between the control group and infected larvae pre-inoculated with yeasts (Dh97 + Va or Yl242 + Va group).*

## Discussion

The host microbiota plays an essential role in its physiology and protection against pathogens. However, this microbiota can be affected by environmental factors, such as bacterial infections. Understanding the dynamic of the microbiome under these disturbances could help to identify new strategies to enhance its resistance and resilience capacity. In this context, some microorganisms, such as probiotics, protect the hosts against pathogen infection; however, their role in the modulation or reinforcement of the microbiota has been less explored with next-generation sequencing. The study of host-microbiota-pathogen interactions in a controlled environment, under a biological context, has been possible using animal models. In the same way, the use of zebrafish as a model organism offers multiple comparative advantages. As a teleostean, zebrafish has an innate and adaptive immune system and its mucosae are colonized by commensal microbiota. In addition, different protocols have been developed to raise them in gnotobiotic conditions and transgenic lines, with specific cell types being fluorescently labeled, allowing the observation of the host responses *in vivo*.

In this study, we explored the preventive effect of two yeast strains (*D. hansenii* 97 and *Y. lipolytica* 242) on the alteration of bacterial microbiome induced by a *V. anguillarum* infection. In addition, we used the transgenic line *Tg(Bacmpx:GFP)*^*i*114^ to analyze the effect of *V. anguillarum* infection on intestinal neutrophil response. Unexpectedly, *V. anguillarum* infection induced a higher mortality rate in *Tg(Bacmpx:GFP)*^*i*114^ larvae than previously described in wildtype Tab5 larvae (Caruffo et al., 2016). The survival rate of conventionally raised *Tg(Bacmpx:GFP)*^*i*114^ larvae at 3 days post infection (8 dpf) was 45%, significantly lower than the 80%, observed in Tab5 larvae (Caruffo et al., 2016). Similar differences occurred in germ-free larvae, where the survival of *Tg(Bacmpx:GFP)*^*i*114^ larvae was 26%, in contrast to the 58% reported before for Tab5 larvae (Caruffo et al., 2016). The pathogen concentration (CFU/larvae) at 8 dpf was almost 1 log_10_ higher in the transgenic larvae compared to those previously observed in Tab5 larvae in conventional and germ-free conditions (Caruffo et al., 2016). We also infected another wildtype strain (Tübingen, TU) to evaluate if the virulence of the pathogen strain could have been increased; observing a higher resistance to *V. anguillarum* infection than *Tg(Bacmpx:GFP)*^*i*114^ larvae (data not shown). These results suggested that differences in the genetic background between the zebrafish lines may be responsible for the variation in the survival rate observed. Additional studies are needed to confirm this hypothesis and elucidate the mechanisms involved. The preinoculation of *Tg(Bacmpx:GFP)*^*i*114^ larvae with *D. hansenii* 97 increased their survival in germ-free and conventional conditions (*p* < 0.05) compared to *V. anguillarum* infected larvae, whereas *Y. lipolytica* 242 did not. The lower colonization capacity (CFU/larvae) of *Y. lipolytica* 242 observed in *Tg(Bacmpx:GFP)*^*i*114^, compared to *D. hansenii* 97, could explain their lower protection effect. This highlights the importance of the dose on probiotic effects, as recently reported (Alvarez et al., 2020). The dose must be adjusted not only considering the probiotic strain but also the host target and disease context.

The main knowledge about the effect of yeasts on the immune response has been made through infection with pathogenic strains. However, the interaction of commensal yeasts and the immune system of vertebrates has been less explored. Yeasts hold β-glucans, mannoproteins, and chitins, which can interact with and stimulate the immune system (Navarrete and Tovar-Ramírez, 2014). Here, we explored the capacity of commensal yeasts to modulate neutrophil response in an infection. Neutrophils are the first immune cells to be recruited to the infection site (Harvie and Huttenlocher, 2015). Therefore, the use of *Tg(Bacmpx:GFP)*^*i*114^ larvae in which neutrophils are specifically fluorescently labeled allows us to evaluate their migration to the affected area, and thus recognize the induction of an immune response to pathogens. As expected, larvae challenged with *V. anguillarum* showed a higher number of neutrophils in the intestine compared to non-infected larvae. Interestingly, infected larvae preinoculated with both yeasts showed less neutrophils than infected larvae, in germ-free condition; although only *D. hansenii* 97 protected larvae from infection. In conventional conditions, we did not observe an anti-inflammatory effect of the yeasts. These observations suggest that, in this infection model, modulation of neutrophil response is not necessarily associated with protection or survival rates. Another important fact to consider in the neutrophil response to yeasts or *V. anguillarum* is the presence of the microbiota in conventional condition. It has been reported that the neutrophil response to a multi-species community cannot be predicted from the relative abundance of its components because a numerically minor member can dominate the host response in some cases (Rolig et al., 2015). Then, it is important to highlight that the neutrophil response of the host to yeasts and/or the pathogen could be, certainly, influenced by its microbiota composition and the immune stimulatory effect of each member. This phenomenon is more relevant at early development stages when microbiota composition could be less stable and more influenced by stochastic and environmental factors (Stephens et al., 2015; Burns et al., 2016).

Further, we evaluated whether pre-inoculation of larvae with *D. hansenii* 97 or *Y. lipolytica* 242 prevents pathogen perturbation of the larval microbiome. It is worthy to note that our conventional experiment was performed in a sterile zebrafish E3 medium and larvae were not fed, therefore, all microorganisms identified in the larval microbiota must have originated from egg chorions. *V. anguillarum* affected the beta diversity of the gut microbial communities significantly but did not alter the alpha diversity and the core microbiota. The relative abundance of *Vibrio*, *Rhizobium*, *Acidovorax*, *Delftia*, *Stenotrophomonas*, *Novosphingobium*, *Ensifer*, *Asticccaulis*, and *Vogesella* was higher and *Acinetobacter* was lower, compared to control. Indeed, *Ensifer*, *Vogesella*, and *Vibrio* were identified as negative predictors for survival. This result suggests that larval survival could be predicted by the abundance of some key genera, rather than the modification of microbial diversity. Several authors show that infections can induce disturbances in the composition of commensal microbiota such as overgrowth of some microorganisms and reduction of others (Khan and Chousalkar, 2020; Vakili et al., 2020; Wang et al., 2020). For example, *V. anguillarum* affected the relative abundance of 18 OTUs (Operational Taxonomic Units) and altered alpha and beta diversity (Nie et al., 2017) in ayu (*Plecoglossus altivelis*). The infection of adult zebrafish (*D. rerio*) with *Streptococcus agalactiae* caused an increase of *Cetobacterium*, *Aeromonas*, *Streptococcus*, and *Plesiomonas*, while *Sphingomonas* decreased (Zhang et al., 2019). In yellow croaker (*Larimichthys crocea*) infected with *Pseudomonas plecoglossicida, Gammaproteobacteria* increased whereas *Actinobacteria* reduced, affecting the abundance of 18 dominant (OTUs) (Li et al., 2020). However, it is important to identify which of these bacterial modifications could explain pathological phenotypes.

In our study, *Ensifer, Vogesella*, and *Vibrio* were predictors of larval mortality. *Vogesella* spp. are aerobic Gram-negative bacteria generally isolated from freshwater sources. The pathogenic potential is controversial, and it is sensitive to some infection perturbation. Moreover, *Vogesella* has been recently isolated from moribund freshwater Nile Tilapia (*Oreochromis niloticus*) (Preena et al., 2020); whereas its abundance decreased in the microbiota of the freshwater snail *Biomphalaria glabrata* infected with the helminth parasite *Angiostrongylus cantonensis* (Osório et al., 2020). The abundance of several taxa, including *Vogesella* and *Ensifer* was significantly associated with helminth parasite *Pseudocapillaria tomentosa* exposure in adult zebrafish, and *Ensifer* was one of the genera that accurately predict exposure (Gaulke et al., 2019). The sequencing of a segment of the 16S rRNA gene (V3–V4 region) does not allow the identification of taxa at the species or strain level. Hence, we could not identify specific species or strains within *Ensifer, Vogesella*, and *Vibrio* genera associated with larval mortality.

Probiotics have been claimed to restore the balance of the intestinal microbiota following a dysbiosis caused by pathogen infection. However, several studies failed to report any impact of probiotics on the microbiota (McFarland, 2014; López Nadal et al., 2020; Wieërs et al., 2020). In contrast to spontaneous recovery, probiotics can delay a post-antibiotic microbiome reconstitution (Suez et al., 2018). Modulation of bacterial microbiota by probiotics has been poorly studied by next-generation sequencing, and few studies have been performed with probiotic yeasts. The co-administration of *Saccharomyces cerevisiae* CNCM I-745 and amoxicillin-clavulanate reduced the overgrowth of *Escherichia* and attenuated the modification in the relative abundance of some other genera induced by the antibiotic in humans (Kabbani et al., 2017). In our study, the effect of probiotic yeasts on microbiota perturbation seems to be selective. We pre-inoculated larvae with two yeasts and then challenged them with the pathogen. The supplementation with each yeast was performed with a single inoculum administered a day before the *V. anguillarum* infection, due to the protective effect of this dosification on larval survival (Caruffo et al., 2016), in comparison with other studies where probiotics were administered for several days. Interestingly, pre-inoculation of infected larvae with yeasts prevented the increase of *Ensifer, Vogesella*, *Novosphingobium*, *Asticcacaulis*, and *Acidovorax* induced by the pathogen. This result suggests that protection of larvae can be associated with modulation of key genera, avoiding the increase of *Ensifer* and *Vogesella*, which are predictors of larval mortality. Inexplicably, we did not identify any positive bacterial predictors for survival which could be further used as candidates to recapitulate protection against this infection, as previously reported (Stressmann et al., 2020).

On the other hand, *D. hansenii* 97 and *Y. lipolytica* 242 induced modification of the beta-diversity of microbiota of non-infected control larvae. This result is similar to those observed in rodents (Albuquerque et al., 2019; Briand et al., 2019) and broilers (Qin et al., 2018), where microbiota of healthy hosts was modulated by some strains of *S. boulardii.* In contrast, the microbiota of healthy humans supplemented with *S. cerevisiae* CNCM I-745 was not affected (Kabbani et al., 2017). The mechanisms involved in the modulation of microbiota by yeasts, in healthy or infected hosts, are largely unknown. Some yeast molecules, such as glycoproteins and polyamines, could be involved (Gómez-Gallego et al., 2012; Wu et al., 2020) alongside other direct (i.e., competition for nutrients and production of antibacterial compounds) or indirect mechanisms (i.e., stimulation of the immune system) (Hatoum et al., 2012).

The inference of the metabolic pathways in bacterial communities through its 16S rRNA gene data is a method commonly used. Among the main advantages of this approach are its low cost and the proven good correlation with genomic content (Langille et al., 2013); the main limitations are that some bacterial families have similar 16S regions despite their difference in genotypes and phenotypes, horizontal gene transfer and the fact that even if a gene is present, it could not be expressed (Langille et al., 2013; Knight et al., 2018). Through this method, the composition of metabolic processes within the control samples was identified. Our results were coherent with previous reports in zebrafish, using PICRUST inferences, and showed a predominance of genes from pathways related to carbohydrate metabolism (Koo et al., 2017; Ma et al., 2020), amino acid metabolism, and membrane transport (Ma et al., 2020). Although no previous studies addressing a characterization of core metabolic pathways within the gut microbiota of zebrafish were found, it is important to emphasize that different studies had shown consistent outputs, even when they used diverse approaches for the analysis, such as PICRUST in the cited literature or Piphillin in this work. Even though the effects of *D. hansenii* in metabolic pathways of the host fishes were previously explored (Angulo et al., 2017), so far, no study has investigated the effects of *D. hansenii* and *Y. lipolytica—*or other yeasts*—*on the metabolic functionality of the host microbiota. However, previous research performed on a metagenomics analysis in the gut microbiota of volunteers consuming Supherb Bio-25, a commercially available probiotic including different bacterial strains, found differences in metabolic pathways of the microbiome specific to person and gut region (Zmora et al., 2018). Nevertheless, consensus is still far from being reached on the effects of probiotics on the metabolism for the host or its microbiota. This highlights the insufficient knowledge of the field, especially in terms of probiotic yeasts. Therefore, further experimental test should be performed to explore their relevance to the protective effect of the studied yeasts. With that in mind and considering the inferential nature of the pathways here discussed, it is imperative to integrate different multi-omic approaches, especially metabolomics, in order to propose mechanistic explanations to our findings.

In general, our study showed that *V. anguillarum* infection, as well as yeast inoculation, modified the beta diversity, abundance of some bacterial genera, and metabolic pathways of the zebrafish larval microbiome. Both yeasts prevented modification of key genera, which were negative predictors of larval survival and some metabolic pathways. These results suggest that probiotic yeasts can interact with the larval microbiome to increase their survival against *V. anguillarum* infection. However, further studies are needed to elucidate the mechanisms involved in the yeasts–pathogen–microbiota interactions, their efficacy in fed larvae, and to evaluate if they are involved in protection against other pathogens.

## Data Availability Statement

The datasets generated for this study can be found in online repositories. The names of the repository/repositories and accession number(s) can be found below: https://www.ebi.ac.uk/ena, PRJEB38908.

## Ethics Statement

The animal study was reviewed and approved by the local and central Committee for Ethics of Animal Experiments from INTA and University of Chile (FCYT1-18-PN).

## Author Contributions

PN, OV, and MG designed the experiments. OV and MG conducted the experimental procedures. MC and DM performed the bioinformatic and statistical analysis. BV conducted Piphillin analysis. KG, AR-J, MT, BV, CF, and PN analyzed the results. PN, OV, BV, and MG wrote the manuscript. All authors read and approved the final manuscript.

## Conflict of Interest

The authors declare that the research was conducted in the absence of any commercial or financial relationships that could be construed as a potential conflict of interest.
