# Supplementary material for: Probiotic Yeasts and Vibrio anguillarum Infection Modify the Microbiome of Zebrafish Larvae
Source: Front Microbiol. 2021 Jun 23;12:647977. doi: 10.3389/fmicb.2021.647977 (PMC8260990; doi:10.3389/fmicb.2021.647977)
Supplement: Supplementary file 5 [file Data_Sheet_1.docx]

Supplementary Material

**
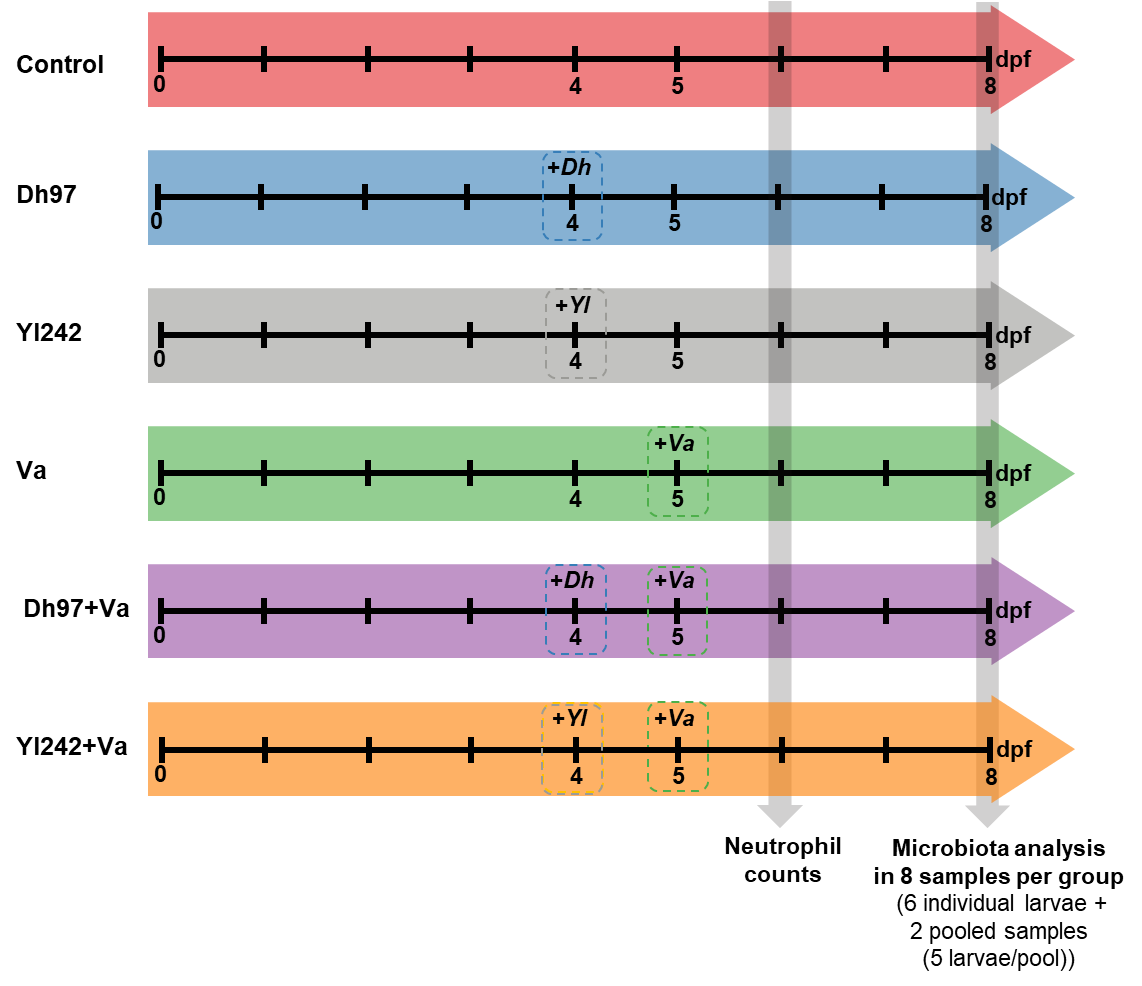
**

**Supplementary Figure S1.** **Experimental design.** *Tg(mpx:GFP)^i114^* zebrafish larvae of 3 days post fertilization (dpf) were divided in 6 groups. **Control group**: conventionally raised larvae (CONV-R). **Dh97 and Yl242 groups**: CONV-R larvae inoculated by immersion with 5 × 10^6^ CFU/mL of *D. hansenii* 97 or *Y. lipolytica* 242, respectively, at 4 dpf. **Va group**: CONV-R larvae challenged at 5 dpf by immersion with 10^7^ CFU/mL of *V. anguillarum*. **Dh97 + Va and Yl242 + Va groups**: CONV-R larvae inoculated by immersion with each yeast at 4 dpf and then challenged with *V. anguillarum* at 5 dpf. Vertical arrows indicate sampling time for neutrophil count (6 dpf) and microbiota analysis (8 dpf). Neutrophil count were also analyzed in germ-free *Tg(mpx:GFP)^i114^* larvae. Survival of germ-free and CONV-R larvae was daily recorded.


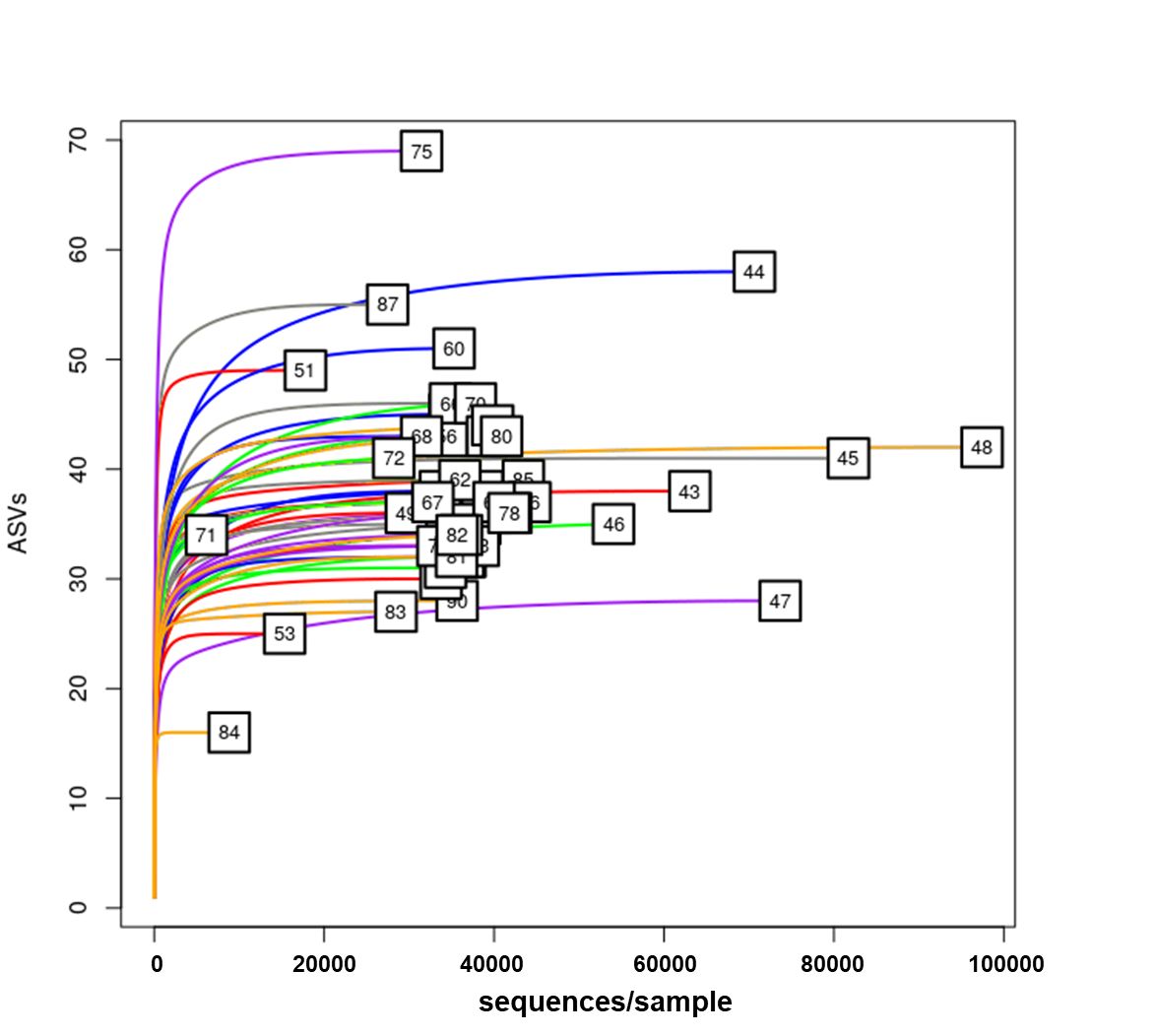


**Supplementary Figure S2. Rarefaction curves for the microbiota of 48 zebrafish larval samples (8 samples per group).** Control group (red curves): conventionally raised larvae (CONV-R). **Dh97 (blue curves) and Yl242 (yellow curves) groups**: CONV-R larvae inoculated by immersion with 5 x 10^6^ CFU/mL of *D. hansenii* 97 or *Y. lipolytica* 242, respectively, at 4 dpf. **Va group** (green curves): CONV-R larvae challenged at 5 dpf by immersion with 10^7^ CFU/mL of *V. anguillarum*. **Dh97 + Va (purple curves) and Yl242 + Va (orange curves) groups**: CONV-R larvae inoculated by immersion with each yeast at 4 dpf and then challenged with *V. anguillarum* at 5 dpf.


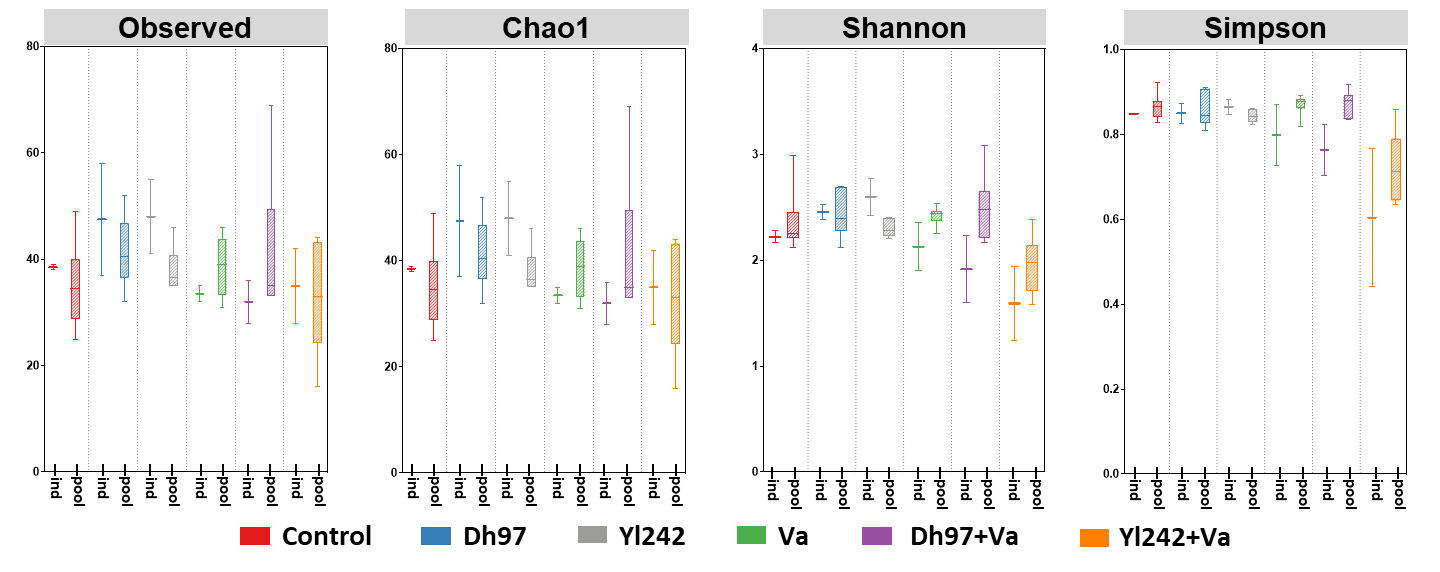


**Supplementary Figure S3. Comparison of alpha diversity of individual (ind) and pooled (pool) samples. Control group**: conventionally raised larvae (CONV-R). **Dh97 and Yl242 groups**: CONV-R larvae inoculated by immersion with 5 x 10^6^ CFU/mL of *D. hansenii* 97 or *Y. lipolytica* 242, respectively, at 4 dpf. **Va group**: CONV-R larvae challenged at 5 dpf by immersion with 10^7^ CFU/mL of *V. anguillarum*. **Dh97 + Va and Yl242 + Va groups**: CONV-R larvae inoculated by immersion with each yeast at 4 dpf and then challenged with *V. anguillarum* at 5 dpf.

**
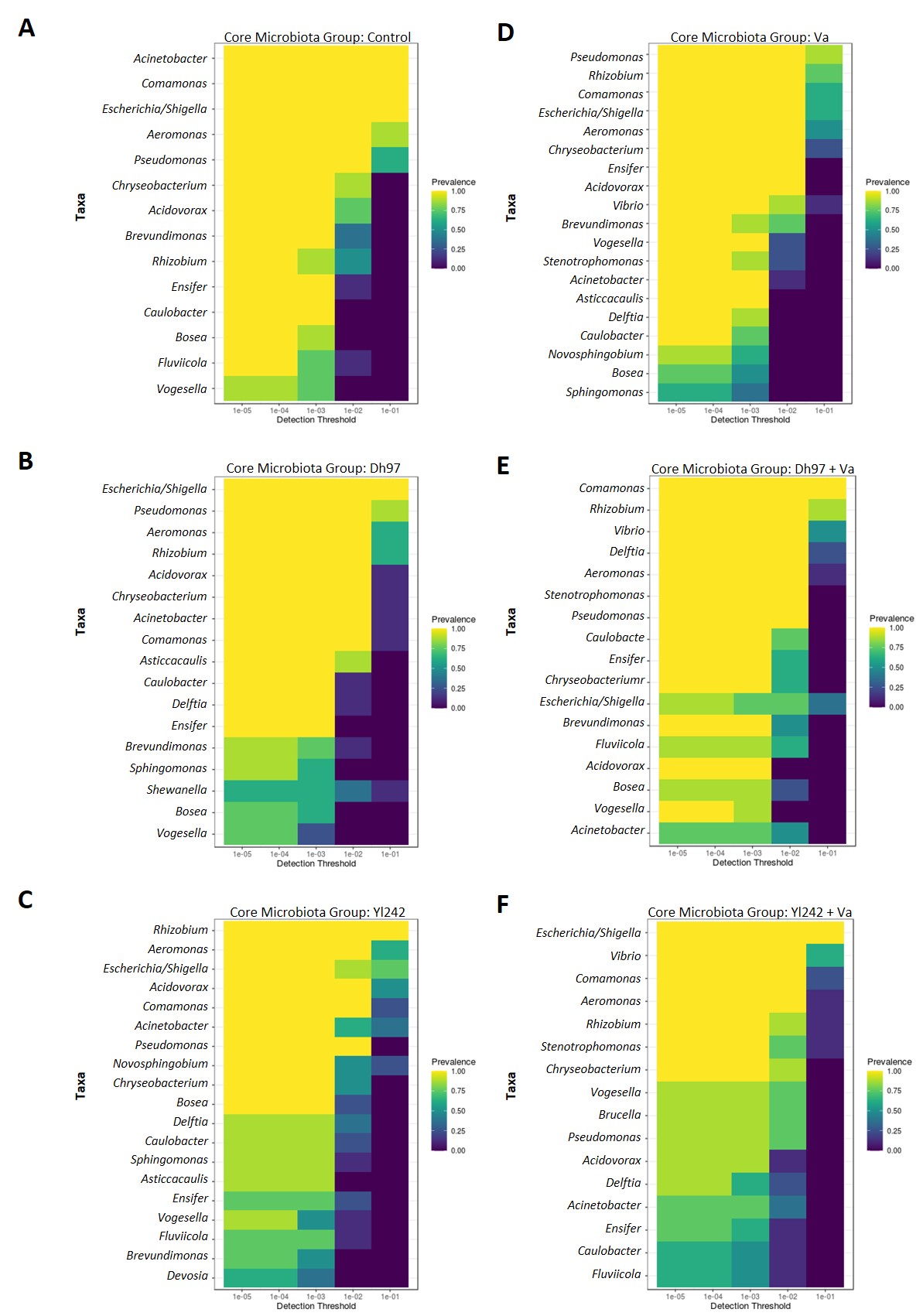
**

**Supplementary Figure S4. Core microbiota. Control group**: conventionally raised larvae (CONV-R). **Dh97 and Yl242 groups**: CONV-R larvae inoculated by immersion with 5 x 10^6^ CFU/mL of *D. hansenii* 97 or *Y. lipolytica* 242, respectively, at 4 dpf. **Va group**: CONV-R larvae challenged at 5 dpf by immersion with 10^7^ CFU/mL of *V. anguillarum*. **Dh97 + Va and Yl242 + Va groups**: CONV-R larvae inoculated by immersion with each yeast at 4 dpf and then challenged with *V. anguillarum* at 5 dpf.

**Supplementary Figure S5. LEfSe analysis identifying taxonomic differences in the microbiota of different groups. Key genera of differently abundant taxa were identified using linear discriminant analysis (LDA) combined with effect size (LEfSe) algorithm. Control group**: conventionally raised larvae (CONV-R). **Dh97 and Yl242 groups**: CONV-R larvae inoculated by immersion with 5 x 10^6^ CFU/mL of *D. hansenii* 97 or *Y. lipolytica* 242, respectively, at 4 dpf. **Va group**: CONV-R larvae challenged at 5 dpf by immersion with 10^7^ CFU/mL of *V. anguillarum*. **Dh97 + Va and Yl242 + Va groups**: CONV-R larvae inoculated by immersion with each yeast at 4 dpf and then challenged with *V. anguillarum* at 5 dpf.

**
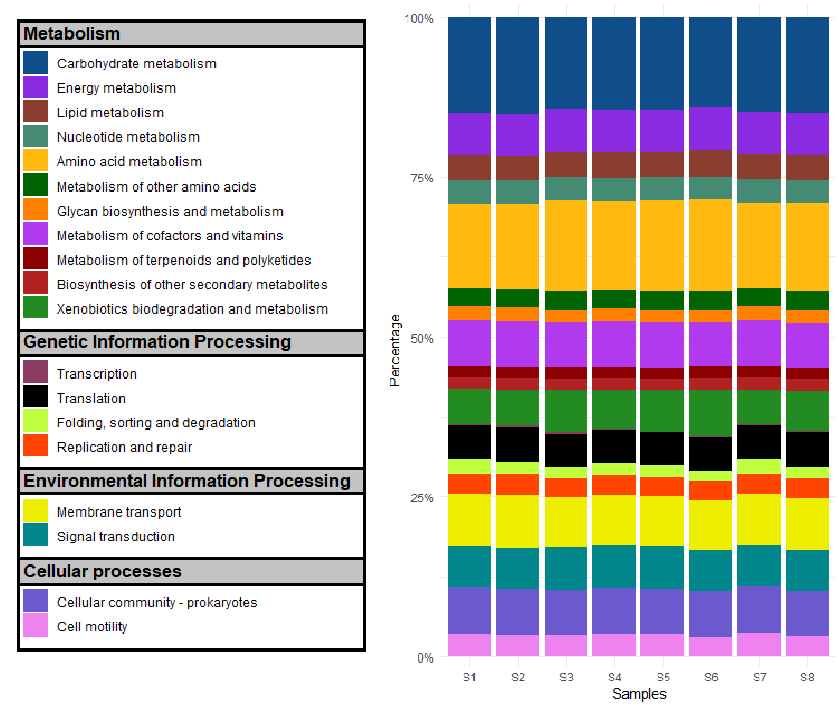
**

**Supplementary Figure S6. Metabolic processes of samples on the control group: conventionally raised *Tg(mpx:GFP)^i114^* zebrafish larvae (CONV-R) at 8 dpf.** All the metabolic processes in the legend correspond to one of the four major KEGG categories used for this analysis. Metabolic pathways were inferred with Piphillin and KEGG database.

**Supplementary Table S1. *V. anguillarum* concentration in conventionally raised (CONV-R) and germ-free (GF) *Danio rerio* larvae.**

|  | ***Vibrio anguillarum* concentration (log_10_ CFU/larva)** | | | | | | | | | | | |
| --- | --- | --- | --- | --- | --- | --- | --- | --- | --- | --- | --- | --- |
| **Group** | **5 dpf** | |  | **6 dpf** | |  | **7 dpf** | |  | **8 dpf** | |  |
|  | **CONV-R** | **GF** |  | **CONV-R** | **GF** |  | **CONV-R** | **GF** |  | **CONV-R** | **GF** |  |
| **Va** | 3.9 ± 0.2 | 4.3 ± 0.4a |  | 4.5 ± 0.4 | 5.6 ± 0.5* |  | 4.9 ± 0.3 | 5.6 ± 0.4* |  | 4.7 ± 0.5^ab^ | 5.2 ± 0.3 |  |
| **Dh97 + Va** | 4.0 ± 0.1 | 3.3 ± 0.6b |  | 4.5 ± 0.1 | 5.4 ± 0.7* |  | 4.9 ± 0.3 | 5.6 ± 0.2* |  | 5.1 ± 0.^1a^ | 5.3 ± 0.5 |  |
| **Yl242 + Va** | 4.0 ± 0.3 | 4.2 ± 0.4ab |  | 4.3 ± 0.3 | 5.7 ± 0.7* |  | 4.7 ± 0.3 | 5.8 ± 0.1* |  | 4.7 ± 0.2^b^ | 5.5 ± 0.2* |  |

Unpaired Mann-Whitney test * indicates significant differences (p ≤ 0.05) between *V. anguillarum* concentrations in CONV-R versus GF. Letters indicate differences (p ≤ 0.05) in *V. anguillarum* concentrations between groups (rows), tested through Kruskal-Wallis test with Dunn multiple comparison corrected test.

**Supplementary Table S2. Yeast concentration conventionally raised (CONV-R) and germ-free (GF) *Danio rerio* larvae.**

|  | **Yeast concentration (log_10_ CFU/larva)** | | | | | | | | | | | | | |
| --- | --- | --- | --- | --- | --- | --- | --- | --- | --- | --- | --- | --- | --- | --- |
|  | **4 dpf** | |  | **5 dpf** | |  | **6 dpf** | |  | **7 dpf** | |  | **8 dpf** | |
| Group | **CONV-R** | **GF** |  | **CONV-R** | **GF** |  | **CONV-R** | **GF** |  | **CONV-R** | **GF** |  | **CONV-R** | **GF** |
| **Dh97** | 4.1 ± 0.2^a^ | 4.5 ± 0.2^a^ |  | 3.0 ± 0.6^a^ | 3.1 ± 0.9 |  | 3.9 ± 0.5^a^ | 4.1 ± 0.5^a^ |  | 3.2 ± 1.0^a^ | 3.9 ± 0.1^a^ |  | 3.2 ± 0.7 | 3.6 ± 0.2^a^ |
| **Yl242** | 2.8 ± 0.2^b^ | 3.1 ± 0.1^b^ |  | 2.6 ± 0.3^ab^ | 3.6 ± 0.3* |  | 2.5 ± 0.8^b^ | 2.8 ± 0.4^b^ |  | 2.3 ± 0.7^ab^ | 3.1 ± 0.4^ab^ |  | 3.2 ± 0.4 | 3.2 ± 0.8^ab^ |
| **Dh97 + Va** | 4.0 ± 0.6^a^ | 4.4 ± 0.5^ac^ |  | 3.3 ± 0.6^a^ | 3.6 ± 0.3 |  | 4.0 ± 0.3^a^ | 3.3 ± 0.3*^ab^ |  | 2.6 ± 0.4^ab^ | 2.7 ± 0.6^b^ |  | 2.2 ± 0.3 | 2.6 ± 0.5^ab^ |
| **Yl242 + Va** | 3.0 ± 0.5^b^ | 3.3 ± 0.4^bc^ |  | 1.6 ± 0.2^b^ | 2.6 ± 0.1* |  | 2.8 ± 0.2^b^ | 2.5 ± 0.5^b^ |  | 1.9 ± 0.1^b^ | 2.2 ± 0.7^b^ |  | 2.3 ± 0.0 | 2.2 ± 0.3^b^ |

Unpaired Mann-Whitney test *indicates significant differences (p ≤ 0.05) between yeasts concentrations in Conv-R versus GF. Letters indicate differences (p ≤ 0.05) in yeast concentrations between groups (rows), tested through Kruskal-Wallis test with Dunn multiple comparison corrected test.

**Supplementary Table S4. Relative abundance of the most abundant taxa in each group.**

|  | **Control** | **Dh97** | **Yl242** | **Va** | **Dh97 +Va** | **Yl242 + Va** |
| --- | --- | --- | --- | --- | --- | --- |
| **Bacterial genera** | Relative abundance mean (%) | | | | | |
| *Acinetobacter* | 21.1 | 5.9 | 8.3 | 0.6 | 1.5 | 0.9 |
| *Comamonas* | 19.5 | 5.5 | 7.2 | 13.6 | 21.8 | 9.0 |
| *Escherichia/Shigella* | 19.1 | 23.4 | 18.5 | 19.4 | 10.6 | 48.9 |
| *Aeromonas* | 18.0 | 11.8 | 16.4 | 10.5 | 7.3 | 5.4 |
| *Pseudomonas* | 11.9 | 21.8 | 3.7 | 18.0 | 5.6 | 2.1 |
| *Chryseobacterium* | 3.8 | 6.1 | 1.0 | 6.2 | 3.2 | 2.9 |
| *Acidovorax* | 1.2 | 6.1 | 9.6 | 4.8 | 0.3 | 0.6 |
| *Rhizobium* | 1.1 | 9.8 | 25.5 | 12.5 | 17.2 | 4.3 |
| *Brevundimonas* | 0.9 | 0.4 | 0.2 | 1.5 | 1.2 | 0.1 |
| *Ensifer* | 0.7 | 0.4 | 0.5 | 4.1 | 1.7 | 0.3 |
| *Fluviicola* | 0.7 | 0.3 | 0.5 | 0.0 | 3.5 | 0.4 |
| *Caulobacter* | 0.6 | 0.7 | 0.6 | 0.5 | 1.9 | 0.3 |
| *Bosea* | 0.3 | 0.2 | 0.9 | 0.1 | 0.7 | 0.0 |
| *Staphylococcus* | 0.2 | 0.0 | 0.0 | 0.0 | 0.0 | 0.0 |
| *Vogesella* | 0.2 | 0.1 | 0.3 | 1.2 | 0.3 | 1.8 |
| *Shewanella* | 0.1 | 2.9 | 0.0 | 0.1 | 0.2 | 0.0 |
| *Brucella* | 0.1 | 0.1 | 0.0 | 0.0 | 0.0 | 3.5 |
| *Vibrio* | 0.1 | 0.0 | 0.0 | 4.5 | 12.0 | 13.0 |
| *Sphingomonas* | 0.0 | 0.3 | 0.5 | 0.1 | 0.1 | 0.2 |
| *Asticcacaulis* | 0.0 | 3.4 | 0.4 | 0.4 | 0.0 | 0.0 |
| *Stenotrophomonas* | 0.0 | 0.1 | 0.0 | 0.9 | 3.7 | 4.6 |
| *Delftia* | 0.0 | 0.5 | 0.8 | 0.5 | 7.0 | 0.6 |
| *Novosphingobium* | 0.0 | 0.0 | 4.3 | 0.3 | 0.0 | 0.0 |
| *Paracoccus* | 0.0 | 0.0 | 0.0 | 0.0 | 0.0 | 0.3 |
| *Salinimicrobium* | 0.0 | 0.0 | 0.0 | 0.0 | 0.0 | 0.3 |
| Other low abundant taxa | 0.4 | 0.3 | 0.5 | 0.2 | 0.3 | 0.3 |

**Supplementary Table S5. Bacterial genera showing significant correlations between larval survival at the end of the experiment (8 dpf) and their relative abundance (Spearman’s correlation analysis).**

| **Bacterial genera** | **Spearman test r** | **p value** |
| --- | --- | --- |
| *Aeromonas* | 0.33197 | 0.0212 |
| *Acinetobacter* | 0.54610 | <0.0001 |
| *Acidovorax* | 0.50104 | 0.0003 |
| *Asticcacaulis* | 0.31768 | 0.0278 |
| *Bosea* | 0.49786 | 0.0003 |
| *Sphingomonas* | 0.34604 | 0.0160 |
| *Devosia* | 0.49820 | 0.0003 |
| *Arsenicibacter* | 0.30516 | 0.0349 |
| *Vibrio* | -0.78516 | < 0.0001 |
| *Chryseobacterium* | -0.31084 | 0.0315 |
| *Stenotrophomonas* | -0.68619 | < 0.0001 |
| *Ensifer* | -0.44392 | 0.0016 |
| *Brevundimonas* | -0.31484 | 0.0293 |
| *Vogesella* | -0.65109 | < 0.0001 |
| *Novospirillum* | -0.30516 | 0.0349 |
| *Xanthomonas* | -0.30516 | 0.0349 |
